# Retraction Note: Shp2 positively regulates cigarette smoke-induced epithelial mesenchymal transition by mediating MMP-9 production

**DOI:** 10.1186/s12931-023-02506-2

**Published:** 2023-08-11

**Authors:** Ya-nan Liu, Yan Guan, Jian Shen, Yong-liang Jia, Jian-cang Zhou, Yun Sun, Jun-xia Jiang, Hui-juan Shen, Qiang Shu, Qiang-min Xie, Yicheng Xie

**Affiliations:** 1 The Children’s Hospital, Zhejiang University School of Medicine, National Clinical Research Center for Child Health, Zhejiang, Hangzhou, 310052 China; 2 Zhejiang Respiratory Drugs Research Laboratory of Food and Drug Administration of China, Zhejiang University School of Medicine, Zhejiang, Hangzhou, 310058 China; 3 The First People’s Hospital of Yancheng, Yancheng, 224001 Jiangsu China; 4 Medical College of Yangzhou University, 11 Huaihai Road, Yangzhou, 225001 Jiangsu China; 5 Sir Run Run Shaw Hospital, Zhejiang University School of Medicine, Zhejiang, Hangzhou, 310000 China; 6 Breath Smooth Biotech Hangzhou Co, LTD, Zhejiang, Hangzhou, 310012 China

**Respiratory Research (2020) 21:161**

10.1186/s12931-020-01426-9

The Editors-in-Chief have retracted this article. Concerns with Fig. 6B were previously addressed with a correction [1] but more image irregularities have been identified, specifically:

Figure 2D: theShp(KO) + CS panel appears to partially overlap with the MK-2206 + CS MMP-9 panel of Fig. 2C in a previously published article [2].

Figure 5A: there appears to be a splice in the Pro-MMP-9 band.

Figure 7A: the JNK 1.25 and 2.5 blots appear to be identical to the JNK //- and +// blots in Fig. 7B.

The Editors-in-Chief therefore no longer have confidence in the results reported in this article.

Authors Ya-nan Liu Jian Shen, Jian-cang Zhou, Hui-juan Shen, Qiang Shu, Qiang-min Xie and Yicheng Xie agree with this retraction. The other authors have not responded to correspondence regarding this retraction.
